# Human and murine APOBEC3s restrict replication of koala retrovirus by different mechanisms

**DOI:** 10.1186/s12977-015-0193-1

**Published:** 2015-08-08

**Authors:** Takayuki Nitta, Dat Ha, Felipe Galvez, Takayuki Miyazawa, Hung Fan

**Affiliations:** Department of Molecular Biology and Biochemistry, University of California, Irvine, Irvine, CA 92697-3905 USA; Cancer Research Institute, University of California, Irvine, Irvine, CA 92697-3905 USA; Department of Biology, Savannah State University, 3219 College St, Savannah, GA 31404-5254 USA; Laboratory of Signal Transduction, Department of Cell Biology, Institute for Virus Research, Kyoto University, 53 Shogoin-Kawaharacho, Sakyo-ku, Kyoto, 606-8507 Japan

**Keywords:** KoRV, APOBEC3, Glyco-gag

## Abstract

**Background:**

Koala retrovirus (KoRV) is an endogenous and exogenous retrovirus of koalas that may cause lymphoma. As for many other gammaretroviruses, the KoRV genome can potentially encode an alternate form of Gag protein, glyco-gag.

**Results:**

In this study, a convenient assay for assessing KoRV infectivity in vitro was employed: the use of DERSE cells (initially developed to search for infectious xenotropic murine leukemia-like viruses). Using infection of DERSE and other human cell lines (HEK293T), no evidence for expression of glyco-gag by KoRV was found, either in expression of glyco-gag protein or changes in infectivity when the putative glyco-gag reading frame was mutated. Since glyco-gag mediates resistance of Moloney murine leukemia virus to the restriction factor APOBEC3, the sensitivity of KoRV (wt or putatively mutant for glyco-gag) to restriction by murine (mA3) or human APOBEC3s was investigated. Both mA3 and hA3G potently inhibited KoRV infectivity. Interestingly, hA3G restriction was accompanied by extensive G → A hypermutation during reverse transcription while mA3 restriction was not. Glyco-gag status did not affect the results.

**Conclusions:**

These results indicate that the mechanisms of APOBEC3 restriction of KoRV by hA3G and mA3 differ (deamination dependent vs. independent) and glyco-gag does not play a role in the restriction.

**Electronic supplementary material:**

The online version of this article (doi:10.1186/s12977-015-0193-1) contains supplementary material, which is available to authorized users.

## Background

Koala retrovirus (KoRV) is a recently discovered retrovirus that infects koalas [1], and it is a likely candidate for the causative agent of lymphomas and other hematologic diseases in these animals. There are several interesting and unique features of KoRV. First, some but not all koalas carry endogenous KoRV-related proviruses, and among different unrelated animals the patterns of endogenous proviral integration sites are distinct [2]. It has also been reported that endogenization of KoRV into koalas is an ongoing process [3]. Thus it appears that KoRV has infected koalas recently, to the extent that individual endogenous KoRV proviruses have not become fixed into the koala population. This contrasts with the situation for endogenous retroviruses in many other species, including humans, where most individual proviruses are shared by all members of the species. Second, KoRV infection appears to be spreading from north to south in Australia, with virtually all animals in the north showing evidence for infection (both endogenous and potentially exogenous KoRVs); in southern regions and on some offshore islands, the incidences of KoRV infection and/or endogenization are lower [4].

KoRV is a member of the gammaretrovirus genus. Gammaretroviruses encode the standard three genes of retroviruses, *gag*, *pol* and *env*, but no accessory proteins such as those of lentiviruses, deltaretroviruses, epsilonretroviruses and betaretroviruses. KoRV’s closest relative is gibbon-ape leukemia virus (GaLV), and it is more distantly related to the murine leukemia viruses (MuLVs) [1]. The predominant KoRV (endogenous and exogenous) is now referred to as KoRV-A, that infects cells by binding to the Pit-1 phosphate transporter as a receptor [5]. Other KoRV isolates (e.g. KoRV-B and others) differ from KoRV-A in the envelope gene [6], and they infect cells via different receptors (e.g. the thiamine transporter for KoRV-B) [7]. It has been suggested that KoRV-B infection is associated with development of lymphoma [7]. KoRV may result from relatively recent cross-species infection of an MuLV-like virus of a southeast Asian rodent into koalas.

We and others have shown that many gammaretroviruses encode an alternate form of Gag polyprotein, glycosylated Gag or glyco-gag [8–10]. We have shown that glyco-gag of Moloney MuLV enhances virus replication by facilitating virus release through lipid rafts [11] and antagonizing a host restriction factor, mouse APOBEC3 (mA3) [12]. Glyco-gags in MuLVs are translated from unspliced viral RNA from an upstream CUG initiation codon in the same reading frame as the AUG codon used for initiation of Gag and Gag-Pol polyproteins. However some endogenous MuLVs (of the class A xenotropic, polytropic and modified polytropic classes) do not have the capacity to encode glyco-gag [13]. The KoRV genome contains an in-frame ORF upstream of Gag characteristic of glyco-gags of exogenous and endogenous MuLVs [13]. In this report, we tested if KoRV expresses a glyco-gag, and if glyco-gag is important for infectivity in vitro. In light of the role of glyco-gag in counteracting restriction by APOBEC3 for M-MuLV we also investigated restriction of wild-type and a putative glyco-gag negative version of KoRV by murine and human APOBEC3s.

## Results

### Use of DERSE cells to detect KoRV infection

**Fig. 1:**
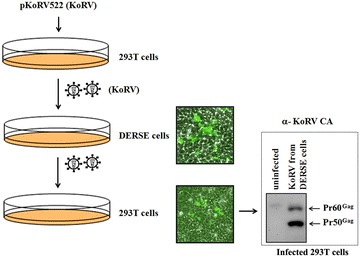
Detection of KoRV infection in DERSE cells. DERSE cells were infected with KoRV released from 293T cells transiently transfected with pKoRV522, and they showed GFP expression as indicated by fluorescence microscopy (*middle* of the figure), indicative of KoRV replication. Supernatants from the infected DERSE cells (after 5 passages) were used to secondarily infect fresh 293T cells (*bottom* of the figure). The infected 293T cells were green and SDS-PAGE and western blot for KoRV Gag protein (*bottom right*) showed that the cultures were expressing KoRV Gag protein, indicative of productive infection. Uninfected 293T and DERSE cells (see Fig. 2) showed no background green fluorescence.

Previous experiments demonstrated that different KoRV isolates can infect human cells through the sodium-dependent phosphate transporter 1 (PiT1) or the thiamine transporter 1 (THTR1) that are present on certain human cell lines, but not on PBMCs [7, 14]. Starting with the infectious KoRV522 clone, we confirmed that KoRV522 can infect cell lines such as human 293T cells and monkey COS7 cells (data not shown). To develop a convenient tool for assessing KoRV infection, we tested KoRV infection of DERSE (Detectors of Exogenous Retroviral Sequence Elements) cells. DERSE cells were originally developed for detecting xenotropic murine leukemia virus-related virus (XMRV) infection [15]. XMRV was initially considered a potential infectious human retrovirus, but it was subsequently found to be a recombinant between two murine endogenous xenotropic retroviruses that occurred during passage of a human prostate cancer in nude mouse xenografts [16, 17]. DERSE cells are derivatives of human LNCaP prostate cancer cells that express an XMRV viral sequence containing an inverted, intron-interrupted GFP reporter cassette. GFP protein is not expressed in DERSE cells, but if they are infected with a replication-competent retrovirus that can package spliced XMRV RNA (with the inverted intron removed) into virus particles, spread of infection to secondarily infected cells results in transcription of the uninterrupted inverted GFP RNA from integrated XMRV DNA, resulting in expression of GFP that can be easily monitored by fluorescence microscopy. Since human cells have functional KoRV receptors, and KoRV is a gammaretrovirus phylogenetically related to murine gammaretroviruses (including XMRV), it seemed possible that KoRV could package XMRV RNA and function in the DERSE cell assay. 293T cells were transiently transfected with the KoRV molecular clone, pKoRV522, and the viruses released from the cells were collected as a source of infectious virus (Fig. 1). Similar to XMRV, DERSE cells infected with KoRV showed individual GFP fluorescent cells 4 days post-infection, indicating that KoRV can package the spliced XMRV-GFP reporter RNA and transfer it to neighboring DERSE cells in a second round of infection (Fig. 1). The infectivity of KoRV released from the infected DERSE cells was also demonstrated by using supernatants from these cells to infect fresh 293T cells (Fig. 1). After serial transfer the infected 293T cells showed strong signals for KoRV Gag protein, indicating that they were also infected with the XMRV-GFP reporter construct (Fig. 1).

**Fig. 2:**
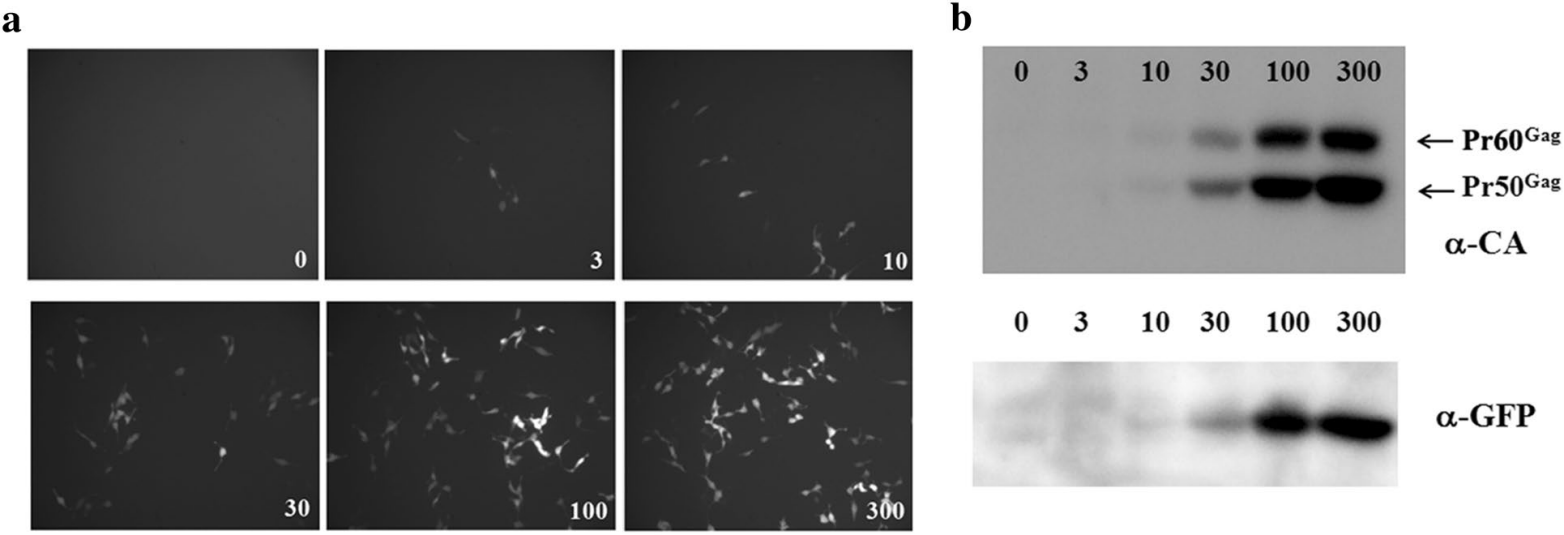
Titration of KoRV infection with DERSE cells. DERSE cells were infected with different doses of KoRV (μl of 293T cell supernatant) as in Fig. 1. Fluorescence for GFP-positive cells is shown in **a**) and SDS-PAGE and western blotting for KoRV Gag and GFP is shown in **b**. The Gag polyprotein precursor Pr60^*gag*^ is indicated, as well as a major proteolytic cleavage product Pr50^*gag*^.

To test if DERSE cells could be used to quantify KoRV infection, the relationship between the amount of KoRV input and levels of GFP or KoRV Gag proteins in infected DERSE cells was assessed. Different amounts of KoRV from 293T cells transiently transfected with pKoRV522 were used to infect DERSE cells. As expected, GFP expression monitored by fluorescence microscopy was correlated with the doses of KoRV (Fig. 2a). In addition, western blots demonstrated that the amounts of KoRV Gag protein and GFP in the infected DERSE cells also were well correlated with the doses of the infecting KoRV (Fig. 2b). These results demonstrated that DERSE cells are a rapid and convenient method to assay and quantify KoRV infectivity.

### Absence of glycosylated gag expression in human cells infected by KoRV

**Fig. 3:**
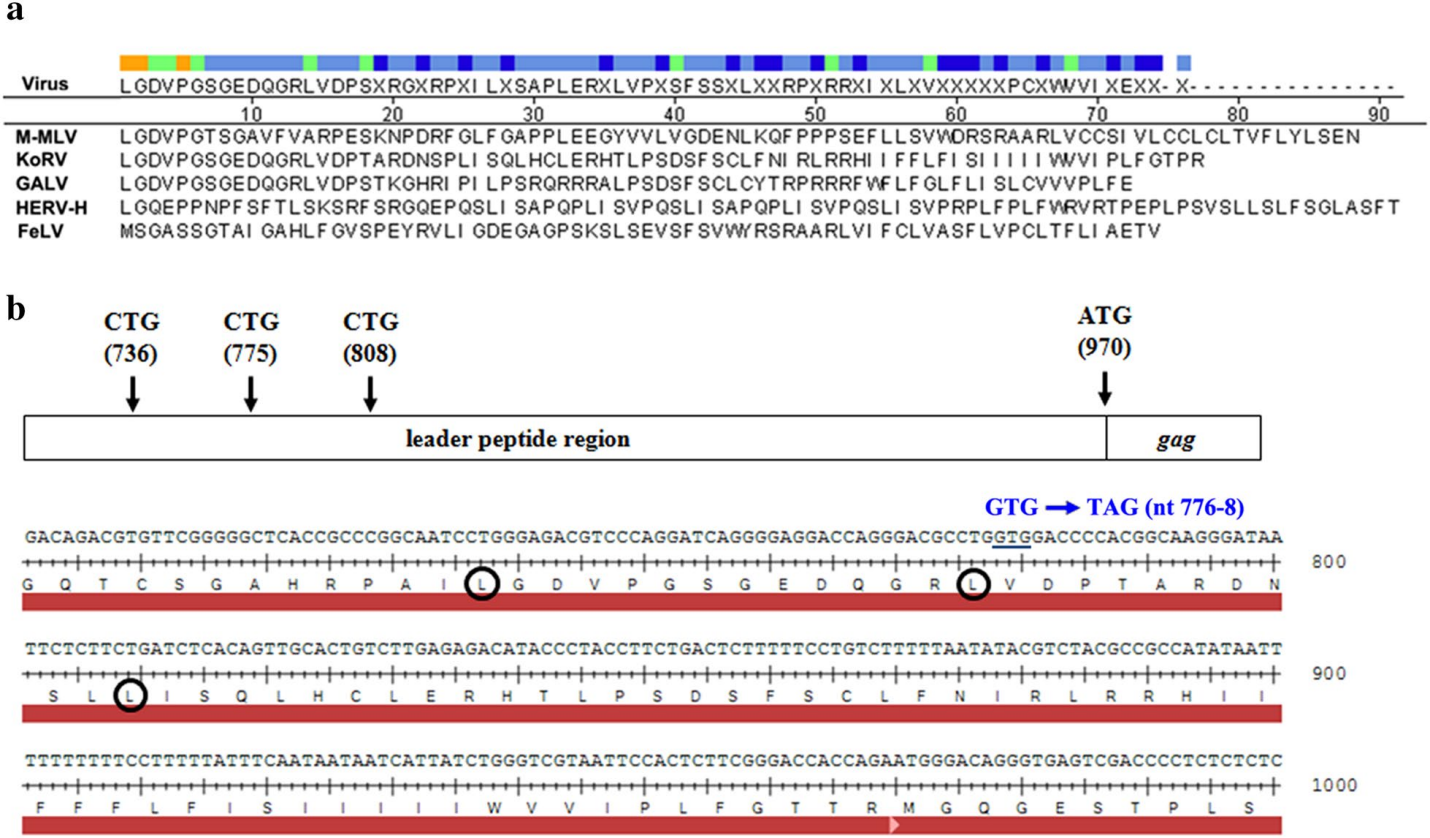
A potential glyco-gag encoded by KoRV. **a** Comparison of amino acid sequences in open reading frames upstream of the AUG for Gag polyprotein is shown for different gammaretroviruses. The sequences are shown assuming initiation at a CUG codon (for leucine), giving maximal sequence identity at the N-terminus. *M-MuLV* Moloney MuLV, *KoRV* koala retrovirus (J group), *GaLV* gibbon ape leukemia virus, *HERV-H* consensus sequence for human endogenous retroviruses of the HERV-H family, *FeLV* feline leukemia virus. A consensus sequence is shown at the* top*, with the degree of identity color coded. There is substantial conservation of the N-terminal amino acids (LGDVP) for all viruses except HERV-H and FeLV; HERV-H shares the two first amino acids of this motif (LG). **b** Amino acid and nucleotide sequences in the region upstream of Pr60^*gag*^ are shown for KoRV (The Pr60^*gag*^ AUG is at nt 970). There are three in-frame CUG (CTG) codons; initiation from the CUG at nt 736 would give the sequence shown in (**a**), with the conserved LGDVP motif. The introduced mutation to generate pKoRV gg- is shown in the **b**.

**Fig. 4:**
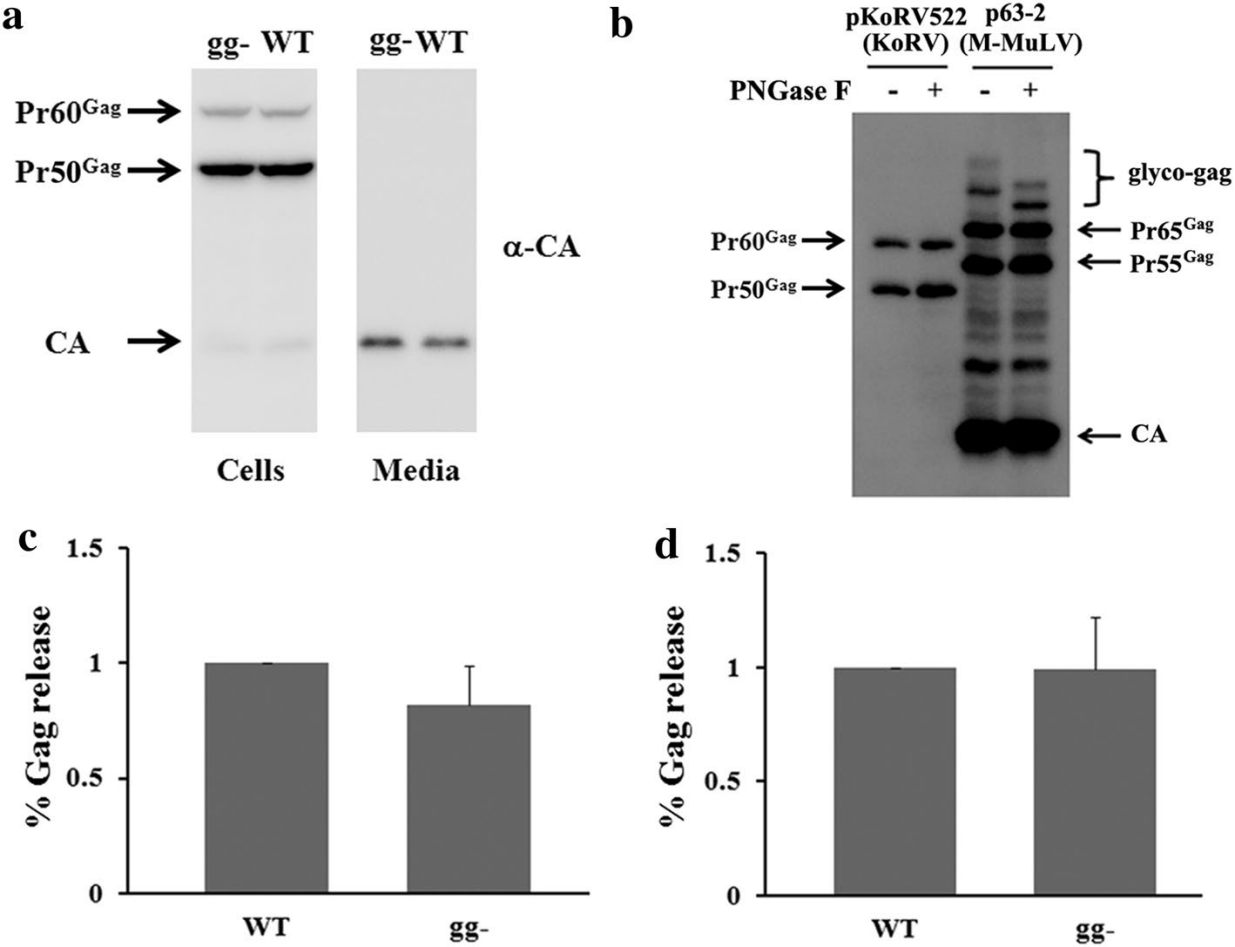
Comparison of WT and putative glyco-gag-mutated KoRVs in viral production. **a** DERSE cells were infected with WT and glyco-gag-mutated (gg-) KoRVs produced from 293T cells transfected with pKoRV522 and pKoRV gg-. Gag in the cell lysates and media were detected by western blots using anti-KoRV CA antibodies. Western blotting for beta-Tubulin in the cell lysates confirmed equal loading of samples (not shown). **b** Cell lysates from 293T cells transfected with pKoRV522 or from the M-MuLV infected cell line 43D were treated with PNGase (endoglycosidase) F to remove N-linked oligosaccharides, and Gag proteins were detected by SDS-PAGE and western blots using anti-KoRV CA and anti-MuLV p30 antibodies. The locations of the Pr65^*gag*^ Gag polyprotein precursor as well as a major cleavage product (Pr55^*gag*^) are shown, as well as the corresponding proteins (Pr60^*gag*^ and Pr50^*gag*^) for KoRV. M-MuLV glyco-gag (the primary translation product gPr80^*gag*^ as well as more slowly migrating forms with additional glycosylation) is indicated; endo F treatment reduced the size of gPr80^*gag*^ to 75 kDa [48]. Virus release efficiencies of gg- KoRV compared to WT KoRV (set at 1 in each experiment) are shown for infected DERSE (**c**) and 293T (**d**) cells. The release efficiency measurements resulted from at least three independent experiments; *error bars* indicate standard deviation.

As mentioned in the introduction, the KoRV genome contains sequences that could potentially encode a typical glyco-gag. Examination of the KoRV (J group) RNA sequence indicated that KoRV has three potential upstream CUG codons in the same reading frame as the Gag polyprotein Pr60^*gag*^ (Fig. 3). Moreover one protein sequence motif conserved in other gammaretroviral glyco-gags is present in the putative glyco-gag of KoRV: LGDVP at the N-terminus if initiation is at the CUG at nt 736. In addition a stretch of hydrophobic (potential membrane-spanning and/or signal peptide) amino acids is immediately upstream of the AUG for Pr60^*gag*^ as for other gammaretroviral glyco-gags. There are three major KoRV isolates with different biological properties (KoRV-A, KoRV-B and KoRV-J) [18], and all of them showed nearly identical nucleic acid and protein sequences beginning with the conserved LGDVP motif in the leader peptide sequence (Additional file 1: Figs. S1, S2). To assess whether KoRV produces functional glyco-gag protein analogous to those in MuLVs, we introduced a mutation that would disrupt expression of putative glyco-gag protein in the plasmid containing the full-length KoRV molecular clone, pKoRV522 (Fig. 3); this plasmid was termed pKoRV gg-. WT and putative glyco-gag mutant KoRV stocks were prepared by transiently transfecting 293T cells with pKoRV522 and pKoRV gg-, and then used to infect DERSE or 293T cells. The infected cells were serially passaged until they all were infected, resulting in the stably infected cells DERSE/WT, DERSE/gg-, 293T/WT and 293T/gg-. As shown in Fig. 4a and quantified in Fig. 4c, the levels of Pr60^*gag*^ in DERSE cells infected with WT and glyco-gag mutant KoRV were equivalent, as were the amounts of CA (virus) released into the media. Likewise 293T cells infected with the two viruses showed equivalent efficiencies of release (Fig. 4d). These results suggested that KoRV glyco-gag may not enhance virus release. On the other hand, western blots using anti-KoRV CA on the WT KoRV-infected cells did not show higher molecular weight proteins in addition to Pr60^*gag*^, which would possibly indicate that the infected DERSE and 293T cells did not express KoRV glyco-gag. In cells infected with WT MuLVs, glyco-gag proteins were sometimes somewhat diffuse in SDS-PAGE due to their glycosylation, and they migrated as more distinct and rapidly migrating bands if the glycosylation was removed [9]. As shown in Fig. 4b, treatment of cell extracts from WT M-MuLV infected cells with PNGase F (endoglycosidase F) showed a shift in mobility of the glyco-gag proteins (gPr80^*gag*^and gPr95^*gag*^-, right lanes). On the other hand, there was no evidence for equivalent proteins in the cells infected with WT KoRV (left lanes). Thus the WT KoRV-infected DERSE and 293T cells did not express glyco-gag.

### Restriction of KoRV infection by APOBEC3 proteins

**Fig. 5:**
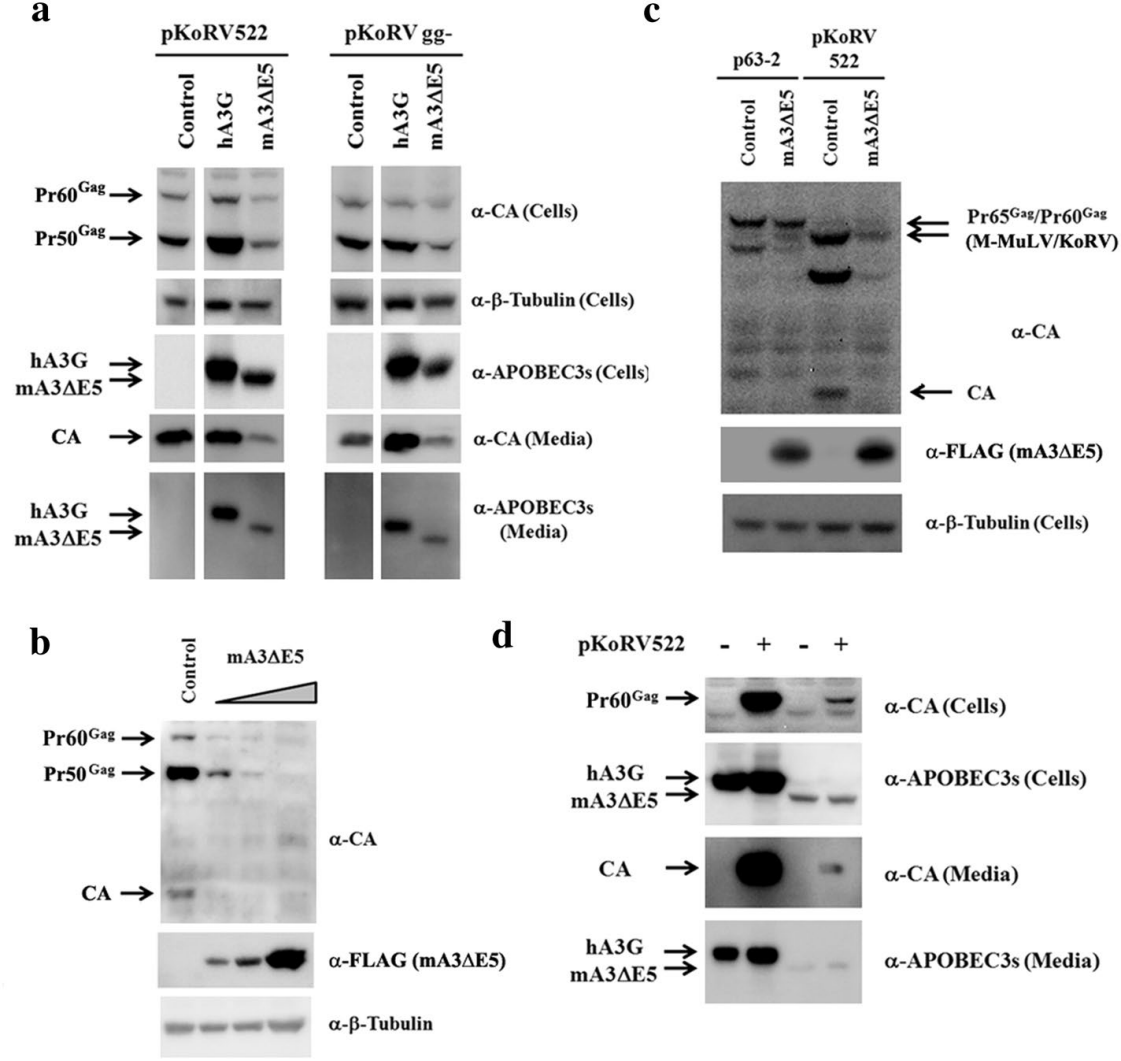
Effects of APOBEC3s on KoRV expression. 293T cells were cotransfected with pKoRV522 or pKoRV gg- along with the plasmids expressing epitope-tagged hA3G or mA3∆E5. **a** KoRV Gag proteins in the cells and media were detected by western blots with anti-KoRV CA. The different APOBEC3 proteins were detected by western blotting with a mixture of antibodies for V5 (hA3G) and FLAG (mA3∆E5). The control and the co-transfected samples shown were from different lanes of the same membranes. **b** Different amounts (ranging from a ratio of 1:0.25 to 1:3) of mA3∆E5 expression vectors were cotransfected with a fixed amount of pKoRV522 (4 µg) in 293T cells. KoRV Gag, mA3∆E5 and beta-Tubulin in the transfected 293T cells were detected by western blots with the appropriate antibodies. **c** The plasmids pKoRV522 (KoRV) and p63-2 (M-MuLV) were cotransfected with the mA3∆E5 expression plasmid into 293T cells at a ratio of 1:1 and Gag proteins were detected by western blot with a mixture of antibodies to the KoRV and M-MuLV CA proteins. **d** 293T cells were transfected with APOBEC expression vectors (hA3G and mA3∆E5) and with pKoRV522. KoRV Gag and APOBECs in the transfected 293T cells and viruses released from the cells were detected by western blots.

APOBEC3 proteins restrict a variety of retroviruses, including HIV-1 and gammaretroviruses. In humans there are multiple APOBEC3 proteins, and APOBEC3G (hA3G) is the predominant restrictor of HIV-1 infection. In mice there is only one APOBEC3 (mA3). We and others have shown that the glyco-gags of Friend and Moloney MuLVs counteract the inhibitory effects of mA3 [12, 19]. Given the phylogenetic relationship between KoRV and MuLVs and the theoretical ability of KoRV to encode a glyco-gag we investigated if (1) hA3G or mA3 can restrict KoRV infection, and (2) if putative KoRV glyco-gag might mediate resistance to these factors. To prepare virus for these experiments 293T cells were transiently transfected with pKoRV522 or pKoRV gg- along with plasmids expressing V5-tagged hA3G and FLAG-tagged mA3∆E5 [a version of mA3 lacking exon 5, present in mouse strains such as C57/BL6] (Fig. 5a). Somewhat surprisingly co-transfection with plasmids expressing mA3∆E5 decreased the amount of KoRV Gag in cells as well as media in dose-dependent manners (Fig. 5b). The effects of mA3∆E5 was observed in 293T cells co-transfected with the pKoRV522 and M-MuLV expression vector p63-2 (Fig. 5c). While MuLV showed minor Gag reduction, KoRV Gag in the co-transfected cells was largely impaired by mA3∆E5. Both hA3G and mA3∆E5 were detected in cells and media, and the signals for mA3∆E5 in media were strong even though released CA was much lower than for medium from cells co-transfected with hA3G. This raised the possibility that some or all of the APOBEC3 proteins released into media from transfected 293T cells could be in non-viral particles (e.g. exosomes) that pelleted through the sucrose cushion during ultracentrifugation. Indeed transfection of 293T cells in the absence of KoRV plasmid resulted in substantial release of both APOBEC3s, indicating substantial fractions of these proteins were released from transfected 293T cells as non-viral particles (Fig. 5d).

**Fig. 6:**
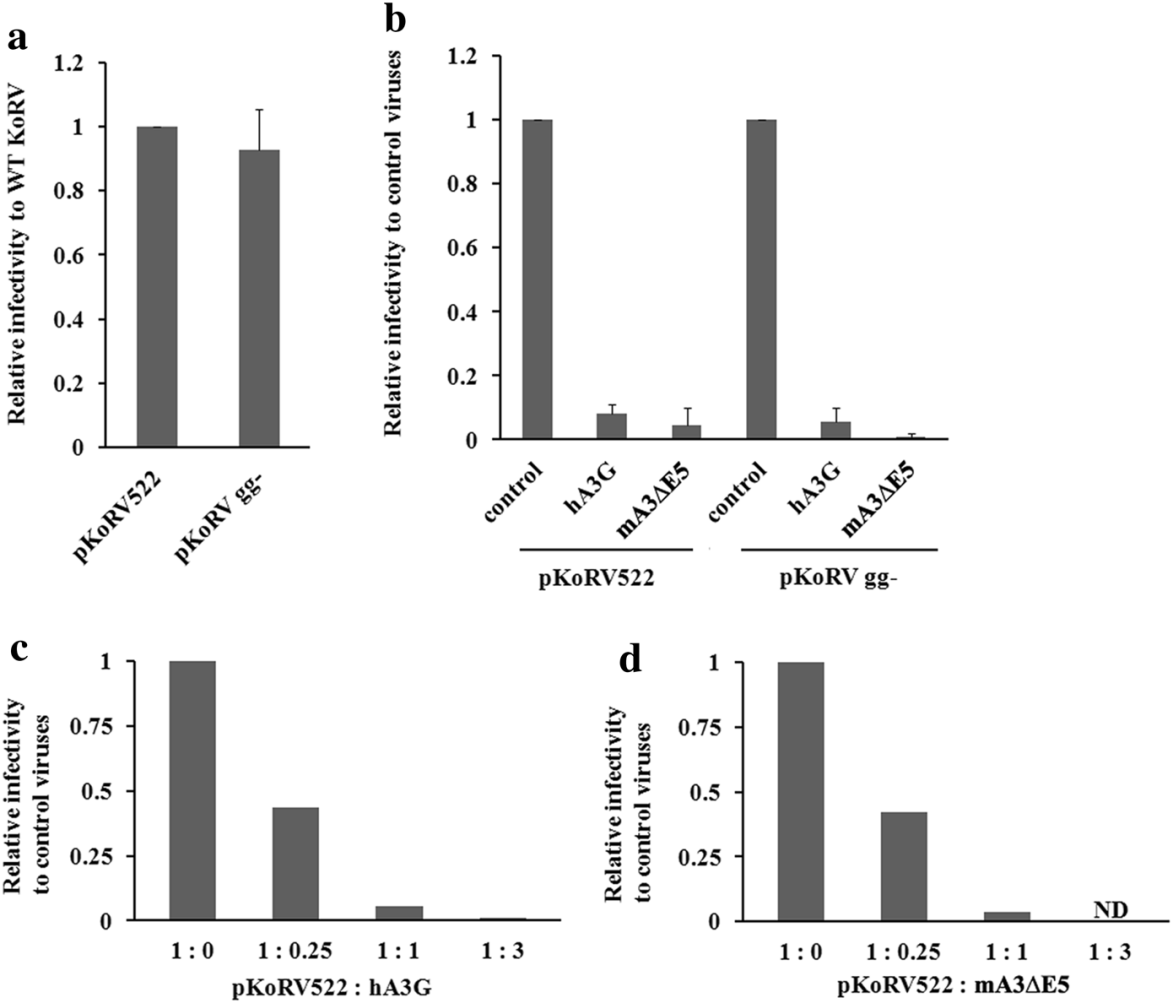
Restriction of KoRV replication by human and mouse APOBEC3s. 293T cells were cotransfected with pKoRV522 or pKoRV gg- along with plasmids expressing hA3G or mA3∆E5. The amount of CA released from the transfected 293T cells was measured by western blots. DERSE cells were infected with the resulting viruses with the similar inputs (measured by KoRV CA) and cell lysates of the infected DERSE cells were subjected to western blots with anti-KoRV CA (Additional file 1: Fig. S3). Relative infectivities of the different KoRV samples were assessed by quantifying the Gag signals by densitometry. The results were normalized for the amounts of the viruses used for infection. All values are shown relative to the infectivity of WT KoRV or KoRVgg- in the absence of any APOBEC3s. The experiments were repeated at least 3 times and the *error bars* indicate standard deviation (**a**, **b**). **a** The relative infectivity of KoRVgg- to KoRV522 is shown, where the infectivity of KoRV522 was set as 1. **b** The effects of co-transfecting different APOBEC3 expression plasmids on the infectivities of WT (pKoRV522) or KoRVgg- virus is shown. The APOBEC3 plasmids were co-transfected into the 293T cells at a 1:1 ratio with the KoRV expression plasmids. Relative viral infectivity to the control virus (without APOBECs) is shown. The experiments in (**a**) and (**b**) were repeated at least three times and the standard deviations are shown. The effects of co-transfecting different amounts of hA3G (**c**) or mA3∆E5 (**d**) with pKoRV522 in the 293T cells on the relative viral infectivity of WT KoRV in DERSE cells is shown. In **d**, it was not possible to obtain enough virus from the co-transfections at a 1:3 ratio for mA3∆E5 for the infectivity assay due to the inhibitory effect of this plasmid on KoRV expression (ND).

To assess the effects of the different APOBEC3s on KoRV, infectivity assays were performed in DERSE cells. The cells were infected with viruses collected from 293T cells transiently transfected with pKoRV522 or pKoRV gg- (to test for the effects of glyco-gag) along with the plasmids expressing APOBEC3s. DERSE cells were infected with media from the different transfections, and levels of infection were determined by SDS-PAGE and western blotting of cell extracts with anti-KoRV CA antiserum. As shown in Fig. 6a, infectivities of WT and gg- KoRVs were comparable. Expression of hA3G and mA3∆E5 strongly impaired KoRV infection (95–98% reduction, Fig. 6b; Additional file 1: Fig. S3). The abilities of hA3G and mA3∆E5 to restrict KoRV infection were dose-dependent and equivalent in strength (Fig. 6c, d). The equivalent restriction of WT and gg- KoRVs by the APOBEC3s was consistent with the lack of detectable expression of glyco-gag in WT KoRV-infected cells (Fig. 4).

The effects of APOBECs on KoRV infection were also examined in cells stably infected with KoRV by transfecting plasmids expressing APOBEC3s into 293T/gg- and 293T/WT cells and measuring the infectivity of virus released from these cells by the DERSE cell assay. Transfection of hA3G and mA3∆E5 into both of these cells showed ca. 40–50% reduction in KoRV infectivity (not shown). These effects were less pronounced than those observed in Fig. 6, where transient co-transfections of KoRV and APOBEC3 expression plasmids were carried out. This may have been due to the fact that the efficiencies of transfection in the 293T/gg- and 293T/WT cells were 50–70%; thus 30–50% of these infected cells would not have expressed the corresponding APOBEC3s. In contrast, in plasmid co-transfections, cells taking up DNA tend to receive and express both plasmids [20].

APOBEC3 proteins are cytidine deaminases. For many retroviruses (e.g. HIV-1 and hA3G), the deaminase activity is important for restriction, resulting in G → A mutations (or hypermutation) in reverse transcribed viral DNA [21]. However hA3G may also restrict HIV infection by deaminase-independent mechanisms. For some other retroviruses (e.g. Moloney MuLV and mA3), restriction is deaminase-independent, and no G → A mutations are observed [22, 23]. Interestingly for M-MuLV, restriction by hA3G results in G → A hypermutation, while mA3 does not; on the other hand, mA3 restriction of HIV-1 results in G → A hypermutation [24, 25]. Thus the mechanisms of APOBEC3 restriction appear to be influenced by both the species of origin of the APOBEC3 protein as well as the particular retrovirus. Both hA3G and mA3∆E5 showed substantial inhibition of KoRV infection as shown in Fig. 6, and we therefore examined if restriction of KoRV by these two APOBEC3s was accompanied by mutations in reverse transcribed DNA. Viruses were gathered from 293T cells transiently transfected with pKoRV522 and hA3G or mA3∆E5 and used to infect DERSE cells. DNA samples were extracted from the infected DERSE cells 36 h post-infection and a portion of KoRV sequence (*pol*-*env* nt 5,637–6,668) was PCR amplified. The PCR products were cloned and individual clones were sequenced to identify mutations in KoRV DNA. As shown in Table 1, KoRV infection in the absence of co-transfected APOBEC3s showed few mutations and no G to A mutations. In contrast 22 out of 38 clones from KoRV/hA3G infected cells contained mutations (99.4% of these mutations were G → A substitutions). On average clones showing mutations had 17.9 mutations (range of 1–55), indicative of hypermutation. Assessment of the local sequence surrounding the G → A mutations (dinucleotide contexts immediately before and after mutations at −1 and +1) demonstrated that 50% of the nucleotides at −1 were T and major target sequences at +1 were G (80%) and A (20%). These results were consistent with previously reported contexts for the G → A mutations induced by hA3G for HIV-1, XMRV and MuLVs [24–27]. In sharp contrast, mA3∆E5 showed few mutations on reverse transcribed KoRV DNA (Table 1), even though inhibition of KoRV infection in DERSE cells by mA3∆E5 was comparable to hA3G (Fig. 6). Thus hA3G and mA3∆E5 restrict KoRV infection by different mechanisms. However the restriction of KoRV by mA3∆E5 did show a higher frequency of clones with mutations than for KoRV by itself, and clones with mutations often showed multiple mutations (average 4.8/clone), indicating weak restriction involving G → A mutation by mA3∆E5.

**Table 1:** hA3G but not mA3∆E5 induces G-to-A hypermutation in KoRV infection

	Clones (No. of mutants)/No. of clones sequenced	No. of bases sequenced	No. of mutations	No. G-to-A mutations (frequency)	No. of bases (bp)/G-to-A mutation	Local sequence +1 (A/T/C/G)	Local sequence −1 (A/T/C/G)
Control	1/24	24,768	1	0 (0%)	n/A		
hA3G	22/38	39,216	393	390 (99.2%)	100.6	51/201/53/85	43/0/1/346
mA3∆E5	4/33	34,056	19	17 (89.4%)	1,792.42	3/4/0/10	6/0/0/11

KoRV viruses were prepared by co-transfecting pKoRV522 along with control, hA3G and mA3∆E5 expression vectors at a ratio of 1:1 into 293T cells. The resulting viruses were infected into DERSE cells and after 36 h DNA was isolated. Sequences from pol-env (AB721500 nt 5,637–6,668) were PCR amplified, cloned and sequenced. The results are compiled in the table.

One potential concern with the results shown in Table 1 could be if multiple PCR products from an individual reverse transcribed viral DNA were cloned and sequenced, which could have led to distortion of the calculated mutation frequencies and over-estimates of the number of bases sequenced. However, for the analysis of KoRV/hA3G DNA, of 23 clones sequenced showing mutations, the mutation patterns for 22 of them were distinct—two clones showed the same pattern and were considered to be from the same original viral DNA. Therefore the data in Table 1 largely represent sequences from different viral DNAs.

## Discussion

In this study, we studied two aspects of KoRV in the context of infectious virus. We first developed an assay system that could conveniently assess KoRV infectivity, through the use of DERSE cells. We then tested if KoRV encodes glyco-gag protein and the results indicated that even though KoRV appears to have the capacity to encode a glyco-gag, it does not do so, at least in human cells. We also tested if APOBEC3 restriction factors can restrict KoRV infection, and found that both hA3G and mA3∆E5 potently restrict KoRV to similar degrees. However while hA3G restriction was accompanied by extensive cytidine deamination, mA3∆E5 restriction was not. Thus restriction of KoRV by these two proteins occurs by predominantly different mechanisms.

DERSE cells were developed as an assay system for infectious retroviruses based on their ability to co-package an XMRV-based RNA containing GFP sequences and transfer it to other DERSE cells by retroviral infection. The fact that KoRV infection of DERSE cells resulted in spread of green fluorescence indicated that KoRV Gag polyprotein can incorporate XMRV RNA into virions, which further suggested that KoRV Gag can recognize the XMRV RNA packaging (Psi) sequence. Indeed there was also extensive spread of both GFP and KoRV Gag to 293T cells infected with the KoRV released from the infected DERSE cells (Fig. 1). Rapid assessments of infectious KoRV can be conducted by western blotting for KoRV Gag or GFP (Fig. 2) or flow cytometry for GFP; highly sensitive quantification could be accomplished by focal immunofluorescence assays. How XMRV RNA interacts with KoRV Gag and/or nucleocapsid protein remains to be determined, but XMRV and KoRV share 59.8% total nucleotide sequence identity (aligned by the Clustal W method, DNASTAR’s MegAlign), so such an interaction is plausible. Likewise alignment of the KoRV sequence with the regions of M-MuLV and XMRV RNAs predicted by mutational and SHAPE analysis [28] to contain their Psi sequences suggests a putative KoRV Psi sequence from nt 565–753 (T. Nitta & H. Fan unpublished). M-Fold predicts a possible stem-loop structure in the center of this sequence, which could be consistent with its function as the KoRV Psi.

One unique feature of gammaretroviruses is that many encode a glyco-gag protein initiated from an in-frame CUG codon upstream of AUG start codon for Gag polyprotein [29]. We and others have shown that MuLV glyco-gag facilitates virus release/assembly [11, 13, 30], protects the reverse transcription complex in viral cores from restriction by mA3 [12], it rescues infectivity of Nef-deficient HIV-1 [31, 32] and it decreases HIV-1 sensitivity to neutralizing antibodies [33]. Phylogenetic comparisons of gammaretroviruses of different species reveal equivalent putative glyco-gag open reading frames with the conserved N-terminal LGDVP motif for viruses ranging from KoRV and Gibbon-Ape leukemia virus (GaLV) through exogenous MuLVs and endogenous gammaretroviruses of mice (XMV class B and C) and humans (HERV-H) [34]. The fact that the glyco-gag open reading frame has been conserved among exogenous retroviruses such as KoRV and GaLV strongly suggests that this protein is expressed and biologically important; its broad distribution among exogenous and endogenous gammaretroviruses suggests that it is an ancient function of these viruses. In this light, it was surprising that the experiments in this study did not detect evidence for expression of glyco-gag by KoRV: (1) neither 293T cells nor DERSE cells productively infected with wild-type KoRV showed glycosylated forms of Gag, (2) mutation of the putative CUG initiation codon for KoRV glyco-gag did not affect virus release efficiency or infectivity, and (3) restriction by both hA3G and mA3∆E5 was efficient and equivalent for WT and KoRV gg-. The pKoRV gg- plasmid contained a stop codon within the LGDVP motif in the putative KoRV glyco-gag coding sequences so it would not encode the most likely form of glyco-gag. In future experiments, it will be interesting to determine the molecular basis for why KoRV does not encode a glyco-gag even though it has the putative coding sequences including three CUG codons.

The apparent lack of glyco-gag expression by KoRV in these experiments might suggest that this protein is dispensable for in vivo infectivity, but on the other hand the conservation of the glyco-gag ORF in KoRV suggests that it is important biologically. One possible explanation could be that KoRV glyco-gag is expressed in cells of its natural host the koala, but not in human cells, which will be interesting to test. Alternatively the maintenance of the putative glyco-gag open reading frame in KoRV could potentially reflect the fact that KoRV glyco-gag is not required for KoRV infection although it was descended from gammaretroviruses (e.g. the GaLV/KoRV progenitor) that do/did express and depend on this protein. KoRV may have been infecting koalas for a relatively short time [35], which may not have been long enough for glyco-gag mutations to have arisen and become fixed in the KoRV genome.

The relationship of glyco-gag and resistance to APOBEC3 restriction for gammaretroviruses is of interest. As described above, glyco-gag is important for resistance of M- and F-MuLV to mA3 in vivo and in vitro [12, 13, 19]. On the other hand, some endogenous MuLVs (XMV class A and the polytropic and modified polytropic PMVs and MPMVs) do not have the capacity to encode a glyco-gag, and sequence analysis suggests that they lost the function [13]. It is striking that endogenous PMV and MPMV proviruses show evidence for G → A mutations, while endogenous XMVs (including class B and Cs that are glyco-gag positive) do not [36]. In fact the replication competent XMRV (derived by recombination between two XMVs) does not encode functional glyco-gag [13]. Interestingly replication of this virus can be potently inhibited by both human and murine APOBEC3s, accompanied by G → A hypermutation [27]. In contrast exogenous M-MuLV is actually relatively resistant to mA3 compared to hA3G; G → A hypermutation is not observed for mA3, but it is for hA3G [23]. This might suggest that glyco-gag is responsible for the differential responses to mA3 and hA3G. In this light the restriction of KoRV infection by mA3 and hA3G was interesting. Both of these factors strongly and equivalently inhibited wt KoRV infection in 293T and DERSE cells, where glyco-gag protein was not detectable. However hA3G restriction was accompanied by extensive G → A hypermutation, while mA3 restriction showed substantially less deamination and hypermutation [although at levels above background (no mA3)]. These results make several points: (1) for KoRV, the mechanisms of restriction are by and large different for hA3G (G → A hypermutation) and mA3 (little mutation), (2) glyco-gag is not determining the response to these factors for KoRV, and (3) some other function or protein of KoRV is responsible for the differential response to mA3 and hA3G. It seems possible that these results may reflect the fact that KoRV was descended from a murine gammaretrovirus, and the murine gammaretroviurses have evolved to replicate in the presence of mA3.

It is noteworthy that mA3 restriction of Moloney and Friend MuLV is accompanied by virtually no G → A mutations, while restriction of Akv MuLV does show measurable mutations [26]. It has recently been reported that this difference maps to the respective glyco-gags of these viruses, and in particular to their levels of glycosylation [37]. It will be interesting to compare the low level G → A mutation observed in mA3 restriction of glyco-gag negative KoRV to that of Akv MuLV.

It is also interesting that marsupials have been reported to lack APOBEC3 genes [38]. The lack of an APOBEC3 gene in koalas would be consistent with the absence of glyco-gag in KoRV, if resistance to APOBEC3 proteins has been a driving force in maintaining glyco-gag during gammaretroviral evolution. As noted in the previous paragraph, the mechanisms of KoRV restriction by hA3G and mA3 may reflect evolution of KoRV from murine gammaretroviruses.

In these experiments, co-expression of mA3 (and hA3B, not shown) decreased the levels of KoRV Gag protein in cells, in addition to inhibiting viral infectivity. The reduction in Gag protein was specific for KoRV, since parallel experiments with M-MuLV did not show this effect. The mechanism(s) of the Gag inhibition remain to be determined, and will be the subject of future experiments. Conceivably this is another mechanism of restriction for these antiviral proteins.

The fact that KoRV can productively infect human cell lines has been described previously and was confirmed here for 293T and DERSE cells. This might suggest concerns for zoonotic infection of humans, particularly in light of the suggestions of KoRV-associated pathologies and malignancies in koalas. However, there have been no reports of transmission of KoRV to humans with significant occupational exposure, e.g. veterinarians or animal keepers [39]. The strong restriction of KoRV by hA3G described here provides support for the low likelihood of zoonotic transmission to humans; hA3G is highly expressed in hematopoietic cells, which are prominent targets for gammaretroviral infection. The 293T and LNCaP cells (from which DERSE cells were derived) that support KoRV replication are known to have little or no hA3G activity [40]. Similar considerations contributed to the conclusions that XMRV is not a human pathogen [41].

## Conclusions

A convenient assay for assessing KoRV infectivity in vitro was employed with DERSE cells. No evidence for expression of a functional glyco-gag in KoRV was observed. KoRV replication was restricted by both mA3 and hA3G regardless of the mutation in the putative glyco-gag sequence. hA3G restriction induced extensive G → A hypermutation during reverse transcription while mA3 restriction did not, suggesting that human and mouse APOBEC3s inhibit KoRV replication in different manners.

## Methods

### Cells and DNA constructs

HEK293T cells [42] were grown in Dulbecco’s modified Eagle’s medium supplemented with penicillin (100 U/ml), streptomycin (100 mg/ml) and 10% fetal bovine serum. DERSE.LiGP (DERSE, Detectors of Exogenous Retroviral Sequence Elements) cells (kindly provided by Dr. Vineet Kewalramani [43] were grown in RPMI1640 supplemented with penicillin (100 U/ml), streptomycin (100 mg/ml), G418 (100 μg/ml) and 10% fetal bovine serum. The full-length molecular clones of KoRV, pKoRV522 [14] and of M-MuLV, p63-2 [44] were described previously. The KoRV mutant, pKoRV gg- that contains a stop codon in the putative KoRV glyco-gag coding sequences (TAG at nt 776–778—NCBI Accession number AB721500) was made by site-directed mutagenesis according to standard techniques [45]. The viral sequence was amplified from pKoRV522 with PfuUltra II Fusion HS DNA Polymerase (Agilent Technology) and the oligomers GACCAGGGACGCCTGTAGGACCCCACGGCAAG and CTTGCCGTGGGGTCCTACAGGCGTCCCTGGTC. The PCR products were digested with DpnI and then transformed with DH5α. The introduced mutation was confirmed by sequencing. The plasmids expressing, V5-tagged hA3G [46] and FLAG-tagged mA3∆E5 [26] were described previously. The plasmids pcDNA3.1(+) and pEGFP-N1 were obtained from Invitrogen and used as controls.

### Antibodies and Chemicals

Rabbit polyclonal anti-MuLVp30^CA^ antiserum [47] and anti-KoRV CA [14] antiserum were described previously. For detection of epitope tags, mouse and rabbit anti-Flag antibodies (Cell Signaling), anti-V5 (Invitrogen) and anti-GFP (Biovision) were used. Beta-Tubulin levels were used to assess sample loading in the gels and were detected by rabbit anti-beta-Tubulin (Cell Signaling). For western blots, we used anti-mouse IgG conjugated with horseradish peroxidase (Thermo Scientific) and anti-rabbit IgG conjugated with horseradish peroxidase (GE Healthcare).

### Detection of viruses and assessment of viral release efficiency

293T and DERSE cells stably infected with wild-type and mutant KoRVs were established by infection with KoRVs produced from 293T cells transiently transfected with pKoRV522 and pKoRV gg-. Detection of infection in the infected cells was by immunofluorescent microscopy for GFP and western blots for KoRV CA protein. The DERSE cells infected with KoRV showed bright GFP fluorescence 6 days post-infection and the fluorescence images were taken with the Axiovert200 microscope (Carl Zeiss). Similarly, GFP in the 293T cells infected with KoRV released from the stably infected DERSE cells was detectable by fluorescence microscopy. For these experiments, the cells were gathered 6–7 days post-infection for detecting KoRV CA protein by western blots. Western blots and assessment of virus release were performed as described previously [11] with slight modifications. In brief, the KoRV-infected 293T and DERSE cells were seeded on 6-cm dishes 1 day before measuring viral release. The media were replaced once and cells were incubated for further 6  h after which both cells and media were gathered. The media were clarified by low-speed centrifugation (10 min at 2,000*g*), passed through a 0.45 μm filter and viral particles were pelleted in a Beckman SW41 rotor at 77,000*g* for 1 h. The pelleted viral particles and corresponding cell lysates were analyzed by SDS-PAGE and western blots using anti-KoRV CA and chemiluminescent detection with an AlphaImager system (Alpha Innotech). To quantify viral release, Pr60^*gag*^, Pr50^*gag*^ and CA bands in the cells and media were quantified with the densitometry software AlphaEaseFC (Alpha Innotech), and the percentage of released Gag divided by total Gag proteins (Pr60^*gag*^, Pr50^*gag*^ and CA) in cells and media was calculated. Different exposures of the blots were analyzed to ensure that densitometry was in the linear range.

### Assessment of antiviral effects of human and mouse APOBEC3s

293T cells were co-transfected with the plasmids expressing KoRVs (pKoRV522 or pKoRV gg-) and APOBEC3s or control plasmids using the CalPhos Mammalian Transfection Kit (Clontech Laboratories). Similarly 293T cells stably infected with KoRVs (293T/WT and 293T/gg-) were transfected with the same APOBEC3 expression plasmids. The media were replaced 6–8 h after transfection and the cells were incubated further for 40 h, after which both the cells and media were harvested. In the experiments using 293T cells stably infected with KoRV, media were replaced again 48 h after APOBEC3 plasmid transfection and the both cells and media were collected 24 h after the second media replacement. Equal portions of the viral and cellular samples were subjected to SDS-PAGE and western blotting for KoRV Gag and APOBEC3s. To evaluate antiviral effects of human and mouse APOBEC3s on KoRV infectivity, DERSE cells were infected with KoRVs released from 293T cells expressing both KoRV and APOBEC3s. A total of 10^5^ DERSE cells were seeded on 6-cm dishes 1 day before infection and the infected cells were gathered 6–7 days post-infection. The cells were lysed by the lysis buffer (50 mM Tris–HCl (pH 8.0), 150 mM NaCl, 0.02% sodium azide, 0.1% SDS, 1% (vol/vol) Nonidet P-40 (NP-40), 0.5% sodium deoxycholate, 20 mM sodium fluoride, 1 mM sodium orthovanadate, and protease inhibitor cocktail (Complete EDTA Free, Roche Diagnostics) and the same volumes of whole cell lysates were subjected to SDS-PAGE and western blots using anti-KoRV CA antibodies. The blots were analyzed by densitometry as described above. The results were normalized for the amounts of the viruses used for infection.

### Assessment of mutations induced by human and mouse APOBECs in KoRV infection

DERSE cells were infected with KoRV prepared from the 293T cells co-transfected with pKoRV522 along with hA3G-V5, mA3∆E5-FLAG or pcDNA3.1. The infected DERSE cells were gathered 36 h post-infection, DNA was extracted, treated with DpnI and a portion of the KoRV *pol* region (NCBI AB721500, nt 5,637–6,668) was PCR amplified using PfuUltra II Fusion HS DNA Polymerase (Agilent Technologies) and the primers 5′-TGCGTCTGGGGAAGTCGTGGG and 5′-CTACTACCGGTGGGGGACTTG. The PCR products were cloned using the Zero Blunt^®^ TOPO^®^ PCR Cloning Kit (Life Technologies) and sequences from individual clones were compared to the original KoRV sequence. PCR products from three or more amplifications were used in two independent infection experiments; the resulting PCR products were cloned into the vector pCR™4Blunt-TOPO^®^ Vector and multiple clones were subjected to sequencing by a commercial vendor (Eton Biosciences).

## Additional file

Additional file 1. Supplemental figures.
