# Supplementary material for: Human and murine APOBEC3s restrict replication of koala retrovirus by different mechanisms
Source: Retrovirology. 2015 Aug 8;12:68. doi: 10.1186/s12977-015-0193-1 (PMC4528783; doi:10.1186/s12977-015-0193-1)
Supplement: Additional file 1. — Supplemental figures. [file 12977_2015_193_MOESM1_ESM.docx]

**ADDITIONAL FIGURES AND FIGURE LEGENDS**

**Fig. S1.** **Comparison of putative glyco-gag nucleic acid sequences among KoRV subgroups.** The nucleotide sequences in the glyco-gag regions for three KoRV subgroups (KoRV-J, -A & -B) were aligned by Clustal Omega (http://www.ebi.ac.uk/Tools/msa/clustalo) [1]. The accession numbers in the NCBI nucleotide database used for the alignment are AB721500.1 (KoRV-J), AF151794.2 (KoRV-A) and KC779547.1 (KoRV-B). *, The nucleotides shared among the KoRVs. The sequences are shown starting with the second CUG from the 5’ end of the RNA (nt 736) that would initiate translation of the putative N-terminal LGDVP motif.

**Fig. S2. Comparison of putative glyco-gag amino acid sequences among KoRV subgroups.** The putative amino acids encoded in the same glyco-gag regions as shown in Supplemental Fig. 1 for the three KoRV subgroups (KoRV-J, -A & -B) were aligned by Clustal Omega as described in Supplemental Fig. 1. *, The predicted glyco-gag amino acids are identical for the KoRVs with two exceptions.

**Fig. S3. Effects of APOBEC3s on KoRV infection. A) 293T cells were cotransfected with pKoRV522 or pKoRVgg- along with plasmids expressing hA3G or mA3ΔE5.** KoRV Gag and APOBECs were detected by western blots. Relative amounts of released virus were quantified by denisitometry (CA in media, left panel) with the densitometry software, AlphaImager system. Equal amounts of the viruses (by using different volumes of viruses from the transfected 293T cells) were used to infect DERSE cells (right panel); the actual relative amounts of virus (determined subsequently by western blots and densitometry for CA of the virus inocula) are indicated as Input on the right panel. Cell lysates of the infected DERSE cells were subjected to SDS-PAGE and western blots with anti-KoRV CA and the levels of infection were assessed from the amounts of KoRV Gag proteins expressed (panel on the right). The relative amounts of Gag protein as measured by densitometry are shown at the bottom of the right panel, and the relative infectivity after correction for the amount of input virus is also shown (Relative infectivity = Relative Gag in the infected DERSE cells / Input). All values are shown relative to the infectivity of WT KoRV or KoRVgg- in the absence of any APOBEC3s (Control). Different exposures of the blots were analyzed to ensure that densitometry was in the linear range. B) A similar independent experiment as shown in panel A. In the upper panels, the amounts of virus produced from transfected 293T cells is shown, and in the lower panel the amount of KoRV Gag proteins produced in infected DERSE cells is shown. Quantification of the input virus and resulting Gag expression was the same as described in A). The data (panels A and B) shown here are representative of the experiments used to calculate the infectivities shown in Fig. 6. For both transfected 293T and infected DERSE cells, all three lanes (Control, hA3G and mA3ΔE5) were from the same western blot; an intervening irrelevant lane is not shown.

**ADDITIONAL REFERENCE**

1. Sievers F, Wilm A, Dineen D, Gibson TJ, Karplus K, Li W, et al. Fast, scalable generation of high-quality protein multiple sequence alignments using Clustal Omega. Mol Syst Biol 2011; 7:539.

To the editor, please replace the current figures S1-3 with low resolution by those with high resolutions. They are attached with emails.
